# Supplementary figures and images for: Metagenomics Analysis of Breast Microbiome Highlights the Abundance of Rothia Genus in Tumor Tissues
Source: J Pers Med. 2023 Feb 28;13(3):450. doi: 10.3390/jpm13030450 (PMC10053322; doi:10.3390/jpm13030450)

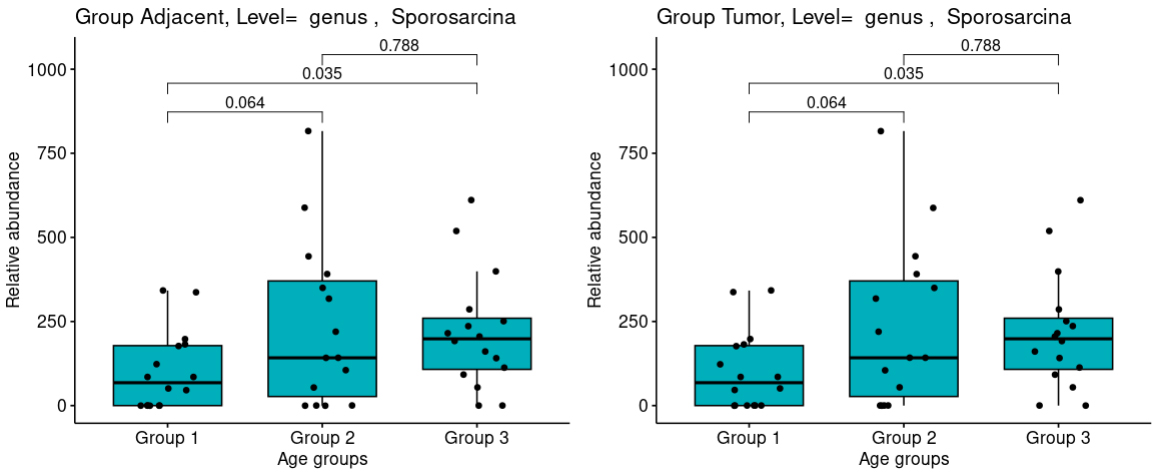

Supplement: Supplementary file 1 [file jpm-13-00450-s001.zip › jpm-2211559-supplementary.jpg]
